# Association of platelet-to-high-density lipoprotein cholesterol ratio and its cumulative exposure with cardiovascular disease risk: a prospective cohort study in Chinese population

**DOI:** 10.3389/fcvm.2025.1580359

**Published:** 2025-05-09

**Authors:** Honglian Luo, Gang Li, Yan Chen, Yun Shen, Wei Shen

**Affiliations:** ^1^ Department of Neurology, Wuhan Fourth Hospital (Wuhan Puai Hospital), Tongji Medical College, Huazhong University of Science and Technology, Wuhan, Hubei, China; ^2^ Division of Population and Public Health Science, Pennington Biomedical Research Center, Baton Rouge, LA, United States

**Keywords:** cardiovascular diseases, platelet-to-high-density lipoprotein cholesterol ratio, cumulative platelet-to-high-density lipoprotein cholesterol ratio, CHARLS, risk prediction, cohort study

## Abstract

**Objective:**

This study aimed to investigate the association of platelet-to-high-density lipoprotein cholesterol ratio (PHR) and its cumulative exposure with cardiovascular disease (CVD) risk.

**Methods:**

The investigation utilized data from the China Health and Retirement Longitudinal Study (CHARLS). Platelet-to-high-density lipoprotein cholesterol ratio was calculated as platelet count (×10⁹/L)/high-density lipoprotein cholesterol (mmol/L), and a cumulative platelet-to-high-density lipoprotein cholesterol ratio (Cumulative PHR) was derived for longitudinal assessment. Multivariable logistic regression models were used to evaluate the association between PHR, cumulative PHR, and CVD risk across three models with increasing adjustments for confounders. Restricted cubic splines (RCS) regressions were utilized to examine if there were non-linear relationships. Subgroup analyses were conducted to enhance the reliability of the study findings. Furthermore, predictive performance was assessed using concordance index (C-index), net reclassification improvement (NRI), and integrated discrimination improvement (IDI).

**Results:**

A total of 7,063 participants aged 45 and older were included, of whom 1,433 (20.29%) experienced a cardiovascular disease. Participants with CVD had higher PHR (167.93 vs. 156.84, *P* < 0.001) and Log PHR (5.12 vs. 5.06, *P* < 0.001) values compared to non-CVD participants. Multivariable logistic regression revealed that Log PHR was independently associated with CVD risk [Odds ratio (OR) per-unit: 1.30, 95% confidence interval (CI): 1.13–1.49, *P* < 0.001; OR per- standard deviation (SD): 1.13, 95% CI: 1.06–1.21, *P* < 0.001]. Log cumulative PHR showed similar associations (OR per-unit: 1.34, 95% CI: 1.05–1.71, *P* = 0.02). Participants in the highest quartile of Log PHR had a nearly 1.32-fold higher risk of CVD compared to the lowest quartile (OR: 1.32, 95% CI: 1.10–1.57, *P* = 0.002). Addition of Log PHR and Log cumulative PHR slightly improved predictive performance metrics of baseline model.

**Conclusion:**

Both Log PHR and Log cumulative PHR are independently associated with increased CVD risk and slightly improved the predictive performance of the baseline risk model. Future research should focus on its clinical implementation and integration into existing risk assessment frameworks.

## Introduction

Cardiovascular diseases (CVD) remain a leading cause of morbidity and mortality worldwide, particularly among middle-aged and older adults (1). In China, the aging population and increasing prevalence of metabolic disorders have contributed to a significant burden of CVD, necessitating improved risk stratification and early detection strategies (2). Traditional risk factors, such as hypertension, diabetes, and dyslipidemia, are well-established contributors to CVD, however, emerging biomarkers that integrate thrombosis, inflammatory and lipid-related components may provide additional predictive value (3–5).

The platelet-to-high-density lipoprotein cholesterol ratio (PHR) has recently been proposed as a novel biomarker reflecting thrombosis, inflammatory, and lipid metabolism status (6–8). Platelets play a crucial role in hemostasis and atherogenesis, while high-density lipoprotein cholesterol is known for its protective cardiovascular effects, including anti-inflammatory and antioxidative properties. Our hypothesis is that an elevated PHR may indicate a prothrombotic and pro-inflammatory state, which could contribute to the development and progression of CVD. Despite its potential clinical relevance, studies examining the association of both the PHR and its cumulative exposure with CVD outcomes are limited, particularly in large prospective cohort studies.

In this study, we aim to investigate the association between the PHR and its cumulative exposure and the incidence of CVD in middle-aged and older Chinese adults using data from the China Health and Retirement Longitudinal Study (CHARLS).

## Methods

### Study design and population

**Figure 1:**
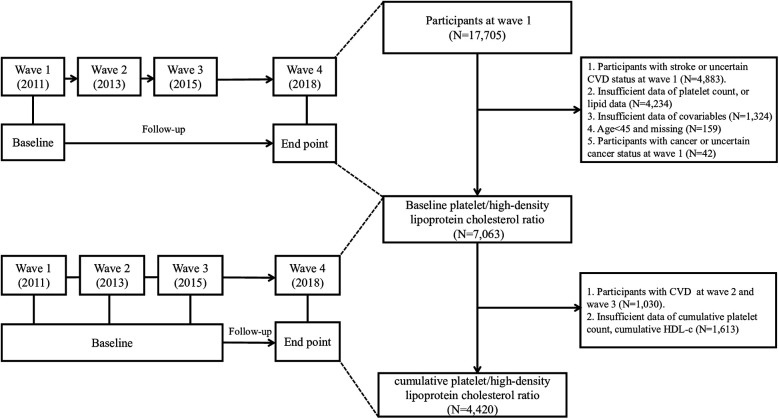
Flow chart of the study participants’ selection process.

The China Health and Retirement Longitudinal Study (CHARLS) is a nationally representative longitudinal survey focusing on Chinese residents aged 45 and older (9). Initiated to address the challenges of an aging population, CHARLS aimed to provide high-quality data on the health, economic, and social conditions of middle-aged and elderly individuals in China. The baseline national survey was conducted between 2011 and 2012, across 150 counties/districts and 450 villages/resident committees. Participants were re-interviewed biennially to track changes over time. In our study, we performed a secondary analysis using data from the CHARLS. Our analysis incorporated data from four waves (2011, 2013, 2015, and 2018). After excluding participants with incomplete follow-up data, pre-existing CVD or cancer, or missing baseline measurements (platelet count, lipid profile and covariates), 7,063 individuals were included in the final cohort. To examine the relationship between cumulative PHR and CVD risk, we identified 4,420 participants who had complete laboratory data (platelet count and lipid profile) at both baseline and third wave (2015), and remained CVD-free during this period. The participant selection process is showed in Figure 1. The study protocol was approved by the Institutional Review Board of Peking University (IRB00001052-11015), and all participants provided written informed consent prior to enrollment.

### Baseline data collection

The CHARLS baseline survey, conducted in 2011–2012, included both self-reported data and objective health measures to ensure high data quality and accuracy. The survey comprehensively captured information across multiple domains through structured data collection modules. Blood samples were collected from participants who provided informed consent. Samples were analyzed for key biomarkers. In this secondary analysis, we extracted data from CHARLS database in terms of age, sex, marital status, education level, residence place, smoking status, alcohol consumption, total cholesterol (TC), triglyceride (TG), BMI, and histories of diseases including hypertension, diabetes, dyslipidemia, history of dyslipidemia medication use, chronic lung disease (CLD), chronic kidney disease (CKD). Key laboratory biomarkers in this analysis included platelet count and high-density lipoprotein cholesterol (HDL-c).

### Exposure variables

The exposure of this analysis was the PHR, which was calculated as the plasma platelet count (10^9^/L) divided by the plasma high-density lipoprotein cholesterol level (mmol/L). Since CHARLS was a longitudinal study, we further defined PHR as baseline PHR and cumulative PHR in the analysis. Cumulative PHR was calculated as Cumulative PHR = [(PHR2012 + PHR2015)/2] × (2015 − 2012) (10–12).

### Ascertainment of incident CVD events

The primary outcome of our study was the incidence of new-onset CVDs. CVD status was determined based on self-reported physician diagnoses of heart diseases or stroke. At each follow-up wave, participants were asked whether a doctor had ever informed them that they had experienced a heart attack, coronary heart disease, angina, congestive heart failure, or any other heart-related condition. Similarly, they were asked whether they had ever been diagnosed with a stroke. Individuals who affirmed a diagnosis of either heart disease or stroke were classified as having CVD. For the cohort examining the association between PHR and CVD, follow-up commenced at wave 1 (2011), whereas for the cohort investigating cumulative PHR and CVD, follow-up began at wave 3 (2015) (Figure 1). Participants were monitored until they experienced their first CVD event or reached the censoring date, whichever occurred first. While self-reported CVD diagnosis has inherent limitations, several validation studies have demonstrated reasonable agreement between self-reported physician-diagnosed diseases and medical record verification in population surveys (13–15). In addition, several previous articles have been published in which CVD was also diagnosed based on questionnaire items in CHARLS (16–22).

### Statistical analysis

Descriptive statistics were used to summarize the baseline characteristics of the study population, stratified by CVD status (non-CVD vs. CVD). Continuous variables were expressed as median (interquartile range) and compared using the Wilcoxon rank-sum test, while categorical variables were presented as frequencies (percentages) and compared using the chi-square test or Fisher's exact test, where appropriate. PHR was subjected to a log transformation and expressed as Log PHR in our analysis due to its non-normal distribution in the original values. First, collinearity among variables was assessed using the variance inflation factor (VIF), with a threshold of VIF < 10 considered acceptable. To evaluate the association between the PHR and CVD risk, multivariable logistic regression models were employed. Three progressively adjusted models were constructed. Model 1 adjusted for age and sex. Model 2 further adjusted for marital status, residence place, education level, smoking status, alcohol consumption, TC, TG, and BMI. Model 3 additionally adjusted for hypertension, diabetes mellitus, history of dyslipidemia medication use, chronic lung disease, chronic kidney disease. PHR was analyzed both as a continuous variable (per-unit and per-SD increases) and as a categorical variable (quartiles, with Q1 as the reference group). The odds ratio (OR) and 95% confidence interval (CI) were reported for each model. A *P*-value for trend was calculated to examine the dose-response relationship across quartiles of PHR. Cumulative metrics of PHR were analyzed using multivariable logistic regression models, following the same adjustment strategy across three models as described above. The OR and 95% CI per-unit, per-SD increases, and quartiles of cumulative PHR were reported.

In addition, restricted cubic spline (RCS) regression models were applied to investigate the nonlinear association of Log PHR and Log cumulative PHR with CVD risk. The overall *P*-value for association and the *P*-value for nonlinearity were calculated to determine whether the relationship between Log PHR, Log cumulative PHR, and CVD risk followed a linear or nonlinear pattern.

To assess the robustness of the findings across different sample characteristics, we conducted subgroup analyses based on age, sex, smoking status, alcohol consumption, BMI, hypertension, diabetes, CLD and CKD. Furthermore, these stratified factors were considered as predetermined potential modifiers of the effects. To evaluate the diversity of the association among subgroups, we incorporated interaction variables and employed likelihood ratio tests.

Furthermore, to evaluate the predictive performance of PHR and cumulative PHR for CVD risk, improvements in predictive ability were assessed using the concordance index (C-index), net reclassification improvement (NRI) and integrated discrimination improvement (IDI) indices (23). Comparisons were made between baseline models and models incorporating PHR or cumulative PHR. All statistical results, including effect sizes, confidence intervals, and *P* values, are reported in accordance with the Declaration of Helsinki and STROBE (Strengthening the Reporting of Observational Studies in Epidemiology) guidelines (24).

All statistical analyses were performed using R software (v4.3.3). A two-sided *P* value <0.05 was considered statistically significant.

## Results

### Baseline characteristics of participants

Table 1 presents the baseline characteristics for the cohort of exploring the association between PHR and CVD, stratified by cardiovascular disease (CVD) status. A total of 7,063 participants were included, with 5,630 (79.71%) in the non-CVD group and 1,433 (20.29%) in the CVD group. Participants with CVD were significantly older than those without CVD (median age: 60 years [54.00, 67.00] vs. 57 years [51.00, 64.00], *P* < 0.001). The platelet-to-high-density lipoprotein cholesterol ratio (PHR) was also higher among individuals with CVD (167.93 [120.66, 226.57] vs. 156.84 [115.71, 210.63], *P* < 0.001), and this difference remained significant when PHR was log-transformed (Log (PHR): 5.12 [4.79, 5.42] vs. 5.06 [4.75, 5.35], *P* < 0.001). There were significant sex differences, with a higher proportion of females in the CVD group (56.80%) compared to the non-CVD group (52.77%) (*P* = 0.006). Marital status, education level and residence place were not significantly different between the groups (*P* = 0.16, 0.07, and 0.64, respectively). Obesity (BMI ≥ 28) was significantly more prevalent in individuals with CVD (16.40% vs. 9.59%, *P* < 0.001). Similarly, hypertension (48.36% vs. 34.44%, *P* < 0.001), diabetes (17.38% vs. 12.38%, *P* < 0.001), and dyslipidemia (45.64% vs. 37.21%, *P* < 0.001) were all more common among those with CVD. Use of lipid-lowering drugs was higher in the CVD group (6.77% vs. 3.06%, *P* < 0.001), as were the prevalences of chronic liver disease (CLD) (12.70% vs. 8.40%, *P* < 0.001) and chronic kidney disease (CKD) (5.86% vs. 4.51%, *P* = 0.04). In addition, baseline characteristics stratified by CVD status for the cohort of exploring the association between cumulative PHR and CVD are presented in Table 2.

**Table 1:** Baseline characteristics stratified by CVD status for the PHR cohort.

Variable	Non-CVD	CVD	*P* value
	*N* = (5,630)	*N* = (1,433)	
Age, years	57.00 (51.00, 64.00)	60.00 (54.00, 67.00)	<0.001
PHR	156.84 (115.71, 210.63)	167.93 (120.66, 226.57)	<0.001
Log (PHR)	5.06 (4.75, 5.35)	5.12 (4.79, 5.42)	<0.001
Total cholesterol, mmol/L	4.91 (4.31, 5.55)	5.01 (4.40, 5.69)	<0.001
Triglyceride, mmol/L	1.16 (0.82, 1.68)	1.24 (0.91, 1.81)	0.03
Sex, *n* (%)			0.006
Female	2,971 (52.77)	814 (56.80)	
Male	2,659 (47.23)	619 (43.20)	
Marital status, *n* (%)			0.16
Married	5,045 (89.61)	1,265 (88.28)	
Other	585 (10.39)	168 (11.72)	
Education level, *n* (%)			0.07
Illiterate	1,592 (28.28)	449 (31.33)	
Junior high school or below	3,572 (63.45)	867 (60.50)	
High school or above	466 (8.28)	117 (8.16)	
Residence place, *n* (%)			0.64
Rural	3,803 (67.55)	958 (66.85)	
Urban	1,827 (32.45)	475 (33.15)	
Smoking status, *n* (%)			0.01
No	3,859 (68.54)	1,032 (72.02)	
Yes	1,771 (31.46)	401 (27.98)	
Alcohol consumption, *n* (%)			<0.001
No	3,646 (64.76)	998 (69.64)	
Yes	1,984 (35.24)	435 (30.36)	
BMI, *n* (%)			<0.001
<28	5,090 (90.41)	1,198 (83.60)	
≥28	540 (9.59)	235 (16.40)	
Hypertension, *n* (%)			<0.001
No	3,691 (65.56)	740 (51.64)	
Yes	1,939 (34.44)	693 (48.36)	
Diabetes, *n* (%)			<0.001
No	4,933 (87.62)	1,184 (82.62)	
Yes	697 (12.38)	249 (17.38)	
Dyslipidemia, *n* (%)			<0.001
No	3,535 (62.79)	779 (54.36)	
Yes	2,095 (37.21)	654 (45.64)	
Lipid-lowering drugs, *n* (%)			<0.001
No	5,458 (96.94)	1,336 (93.23)	
Yes	172 (3.06)	97 (6.77)	
CLD, *n* (%)			<0.001
No	5,157 (91.60)	1,251 (87.30)	
Yes	473 (8.40)	182 (12.70)	
CKD, *n* (%)			0.04
No	5,376 (95.49)	1,349 (94.14)	
Yes	254 (4.51)	84 (5.86)	

**Table 2:** Baseline characteristics stratified by CVD status for the cumulative PHR cohort.

Variable	Non-CVD	CVD	*P* value
	*N* = (3,882)	*N* = (538)	
Age, years	57.00 (51.00, 63.00)	60.00 (54.00, 65.00)	<0.001
Cumulative PHR	472.53 (359.34, 604.62)	489.12 (376.11, 655.79)	0.007
Log (cumulative PHR)	6.16 (5.88, 6.40)	6.19 (5.93, 6.49)	0.01
Total cholesterol, mmol/L	4.90 (4.31, 5.54)	5.08 (4.44, 5.74)	<0.001
Triglyceride, mmol/L	1.16 (0.83, 1.70)	1.23 (0.91, 1.79)	0.10
Sex, *n* (%)			0.01
Female	2,081 (53.61)	320 (59.48)	
Male	1,801 (46.39)	218 (40.52)	
Marital status, *n* (%)			0.95
Married	3,500 (90.16)	484 (89.96)	
Other	382 (9.84)	54 (10.04)	
Education level, *n* (%)			0.12
Illiterate	1,085 (27.95)	173 (32.16)	
Junior high school or below	2,504 (64.50)	325 (60.41)	
High school or above	293 (7.55)	40 (7.43)	
Residence place, *n* (%)			0.48
Rural	2,651 (68.29)	376 (69.89)	
Urban	1,231 (31.71)	162 (30.11)	
Smoking status, *n* (%)			0.01
No	2,661 (68.55)	398 (73.98)	
Yes	1,221 (31.45)	140 (26.02)	
Alcohol consumption, *n* (%)			0.34
No	2,519 (64.89)	361 (67.10)	
Yes	1,363 (35.11)	177 (32.90)	
BMI, *n* (%)			<0.001
<28	3,494 (90.01)	457 (84.94)	
≥28	388 (9.99)	81 (15.06)	
Hypertension, *n* (%)			<0.001
No	2,561 (65.97)	275 (51.12)	
Yes	1,321 (34.03)	263 (48.88)	
Diabetes, *n* (%)			<0.001
No	3,409 (87.82)	445 (82.71)	
Yes	473 (12.18)	93 (17.29)	
Dyslipidemia, *n* (%)			<0.001
No	2,411 (62.11)	285 (52.97)	
Yes	1,471 (37.89)	253 (47.03)	
Lipid-lowering drugs, *n* (%)			<0.001
No	3,756 (96.75)	498 (92.57)	
Yes	126 (3.25)	40 (7.43)	
CLD, *n* (%)			0.001
No	3,564 (91.81)	471 (87.55)	
Yes	318 (8.19)	67 (12.45)	
CKD, *n* (%)			0.15
No	3,722 (95.88)	508 (94.42)	
Yes	160 (4.12)	30 (5.58)	

### The association between the Log PHR and CVD

**Figure 2:**
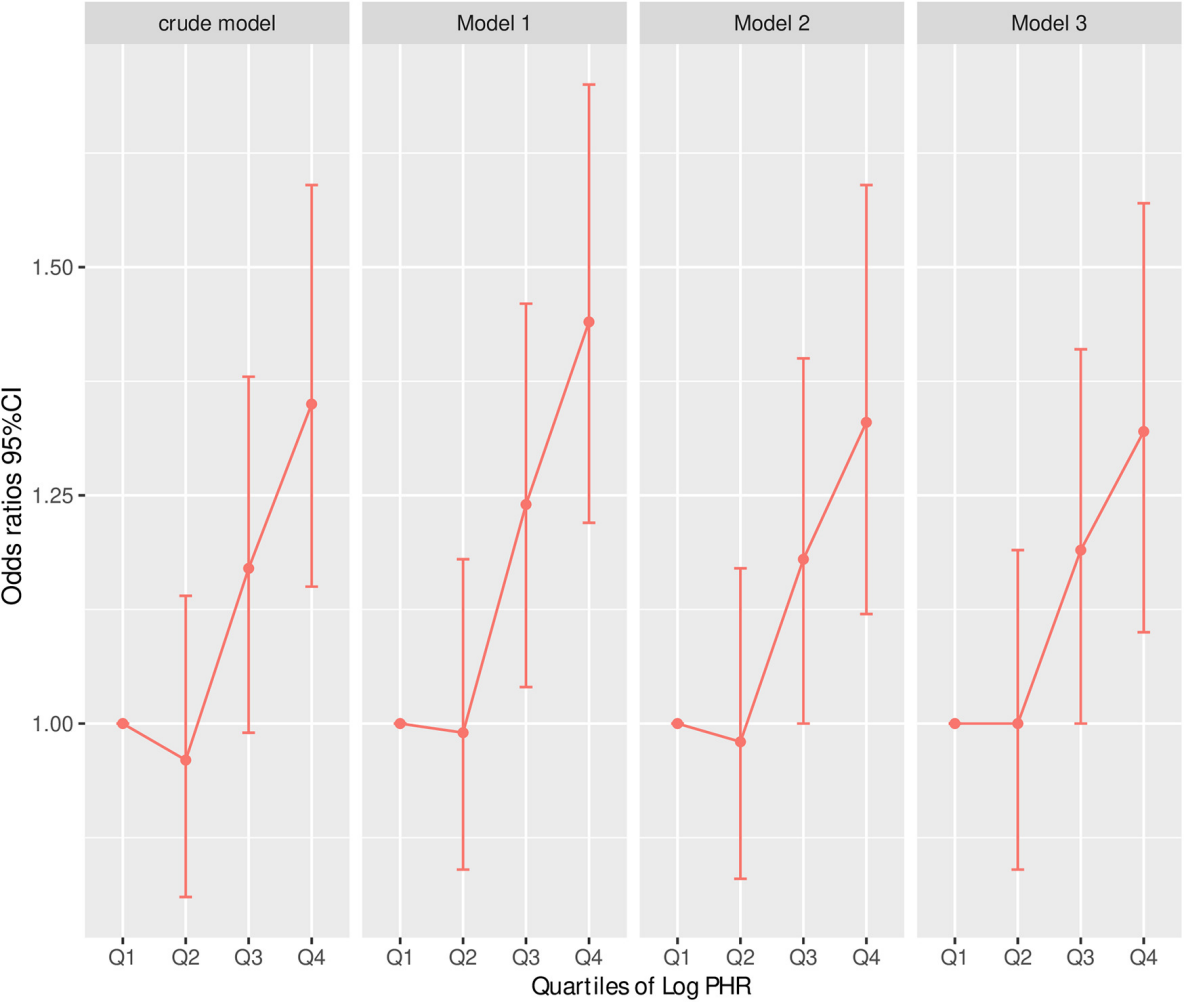
Risk of CVD according to Log (cumulative PHR) quartiles in different adjustment models. Crude model: without adjusting for covariates. Model 1: adjusted for age and sex. Model 2: further adjusted for marital status, residence place, education level, smoking status, alcohol consumption TC, TG, and BMI. Model 3: additionally adjusted for hypertension, diabetes, history of dyslipidemia medication use, chronic lung disease, chronic kidney disease. Odds ratios and 95% confidence intervals are shown, with the lowest quartile (Q1) as reference.

Collinearity diagnostics showed that Variance Inflation Factors (VIF) for all covariates were below the threshold of 10, indicating no significant collinearity (Supplementary Tables S1, S2). Table 3 presents the results of multivariable logistic regression models assessing the association between the logarithm of the platelet-to-high-density lipoprotein cholesterol ratio (Log PHR) and CVD. Across all models, higher Log PHR was significantly associated with an increased risk of CVD. In the per-unit increase analysis, Log PHR was significantly associated with CVD in Model 1 (OR: 1.39, 95% CI: 1.23–1.57, *P* < 0.001), Model 2 (OR: 1.31, 95% CI: 1.14–1.50, *P* < 0.001), and Model 3 (OR: 1.30, 95% CI: 1.13–1.49, *P* < 0.001), even after adjusting for multiple covariates including demographic factors, lifestyle behaviors, lipid levels, and comorbidities. Similarly, in the per-standard deviation (SD) increase analysis, Log PHR remained a significant predictor of CVD, with OR of 1.17 (95% CI: 1.10–1.24, *P* < 0.001) in Model 1, 1.14 (95% CI: 1.07–1.21, *P* < 0.001) in Model 2, and 1.13 (95% CI: 1.06–1.21, *P* < 0.001) in Model 3. When Log PHR was analyzed as a categorical variable (quartiles), individuals in the highest quartile (Q4) had a significantly higher risk of CVD compared to those in the lowest quartile (Q1). The OR for Q4 were 1.44 (95% CI: 1.22–1.70, *P* < 0.001) in Model 1, 1.33 (95% CI: 1.12–1.59, *P* = 0.001) in Model 2, and 1.32 (95% CI: 1.10–1.57, *P* = 0.002) in Model 3. The trend across quartiles was statistically significant in all models (*P* for trend <0.001) (Table 3 and Figure 2).

**Table 3:** Multivariable logistic regression models of the association between Log PHR and CVD.

Log PHR and CVD	Model 1		Model 2		Model 3	
	OR (95% CI)	*P*	OR (95% CI)	*P*	OR (95% CI)	*P*
Per-unit	1.39 (1.23, 1.57)	<0.001	1.31 (1.14, 1.50)	<0.001	1.30 (1.13, 1.49)	<0.001
Per-SD	1.17 (1.10, 1.24)	<0.001	1.14 (1.07, 1.21)	<0.001	1.13 (1.06, 1.21)	<0.001
Log PHR as categories variable (Q1 as reference)						
Q2	0.99 (0.84, 1.18)	0.94	0.98 (0.83, 1.17)	0.83	1.00 (0.84, 1.19)	0.99
Q3	1.24 (1.04, 1.46)	0.01	1.18 (1.00, 1.40)	0.05	1.19 (1.00, 1.41)	0.05
Q4	1.44 (1.22, 1.70)	<0.001	1.33 (1.12, 1.59)	0.001	1.32 (1.10, 1.57)	0.002
*P* for trend		<0.001		<0.001		<0.001

Model 1: adjusted age and sex; Model 2: adjusted age, sex, marital status, residence place, education level, smoking status, alcohol consumption, TC, TG, and BMI; Model 3: adjusted age, sex, marital status, residence place, education level, smoking status, alcohol consumption, TC, TG, BMI, hypertension, diabetes, history of dyslipidemia medication use, CLD, CKD.

### The association between the Log cumulative PHR and CVD

**Figure 3:**
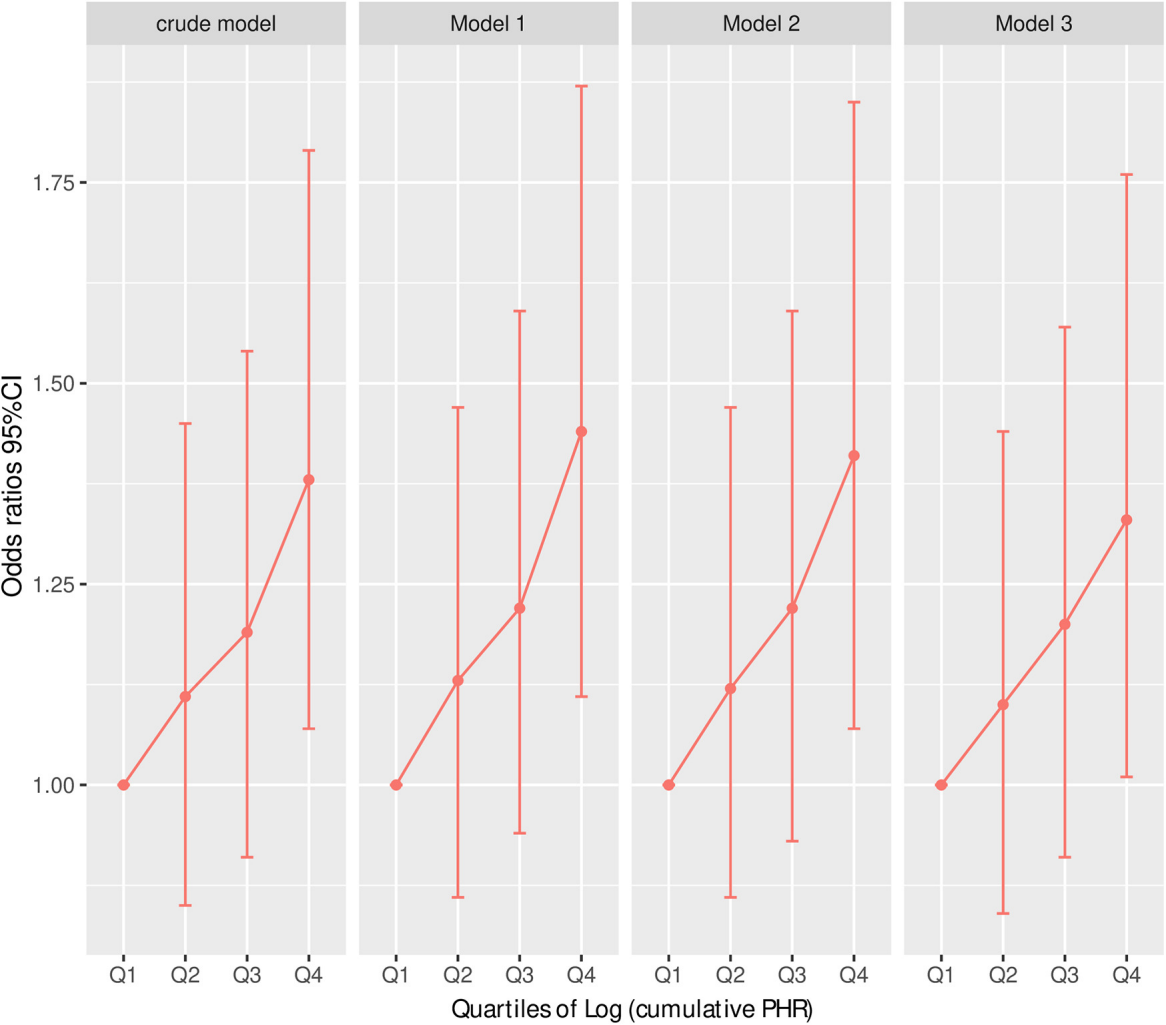
Risk of CVD according to Log (cumulative PHR) quartiles in different adjustment models. Crude model: without adjusting for covariates. Model 1: adjusted for age and sex. Model 2: further adjusted for marital status, residence place, education level, smoking status, alcohol consumption TC, TG, and BMI. Model 3: additionally adjusted for hypertension, diabetes, history of dyslipidemia medication use, chronic lung disease, chronic kidney disease. Odds ratios and 95% confidence intervals are shown, with the lowest quartile (Q1) as reference.

Table 4 presents the results of multivariable logistic regression models examining the association between the cumulative platelet-to-high-density lipoprotein cholesterol ratio (Log cumulative PHR) and CVD. Across all models, higher Log cumulative PHR was significantly associated with an increased risk of CVD. For each unit increase in Log cumulative PHR, the odds of CVD were significantly elevated in Model 1 (OR: 1.40, 95% CI: 1.12–1.76, *P* = 0.004), Model 2 (OR: 1.38, 95% CI: 1.08–1.76, *P* = 0.01), and Model 3 (OR: 1.34, 95% CI: 1.05–1.71, *P* = 0.02), even after adjusting for a range of demographic, lifestyle, and clinical covariates. Similarly, in the per-standard deviation (SD) increase analysis, Log cumulative PHR remained a significant predictor of CVD, with OR of 1.15 (95% CI: 1.05–1.26, *P* = 0.004) in Model 1, 1.14 (95% CI: 1.03–1.26, *P* = 0.01) in Model 2, and 1.13 (95% CI: 1.02–1.24, *P* = 0.02) in Model 3. When Log cumulative PHR was analyzed as a categorical variable (quartiles), individuals in the highest quartile (Q4) exhibited a significantly greater risk of CVD compared to those in the lowest quartile (Q1). The odds ratios for Q4 were 1.44 (95% CI: 1.11–1.87, *P* = 0.01) in Model 1, 1.41 (95% CI: 1.07–1.85, *P* = 0.01) in Model 2, and 1.33 (95% CI: 1.01–1.76, *P* = 0.04) in Model 3. The *P*-trend values were significant across all models (*P* for trend = 0.004, 0.01, and 0.03, respectively), indicating a dose-response relationship between cumulative Log PHR and CVD risk. In contrast, the second and third quartiles (Q2 and Q3) did not show statistically significant associations with CVD, suggesting that the increased risk is primarily driven by those in the highest cumulative Log PHR category (Table 4 and Figure 3).

**Table 4:** Multivariable logistic regression models of the association between Log (cumulative PHR) and CVD.

Log (cumulative PHR) and CVD	Model 1		Model 2		Model 3	
	OR (95% CI)	*P*	OR (95% CI)	*P*	OR (95% CI)	*P*
Per-unit	1.40 (1.12, 1.76)	0.004	1.38 (1.08, 1.76)	0.01	1.34 (1.05, 1.71)	0.02
Per-SD	1.15 (1.05, 1.26)	0.004	1.14 (1.03, 1.26)	0.01	1.13 (1.02, 1.24)	0.02
Log (cumulative PHR) as categories variable (Q1 as reference)						
Q2	1.13 (0.86, 1.47)	0.38	1.12 (0.86, 1.47)	0.40	1.1 (0.84, 1.44)	0.48
Q3	1.22 (0.94, 1.59)	0.14	1.22 (0.93, 1.59)	0.15	1.2 (0.91, 1.57)	0.19
Q4	1.44 (1.11, 1.87)	0.01	1.41 (1.07, 1.85)	0.01	1.33 (1.01, 1.76)	0.04
*P* for trend		0.004		0.01		0.03

Model 1: adjusted age and sex; Model 2: adjusted age, sex, marital status, residence place, education level, smoking status, alcohol consumption, TC, TG, and BMI; Model 3: adjusted age, sex, marital status, residence place, education level, smoking status, alcohol consumption, TC, TG, BMI hypertension, diabetes, history of dyslipidemia medication use, CLD, CKD.

### Exploring the potential nonlinear association of Log PHR, Log cumulative PHR and CVD

**Figure 4:**
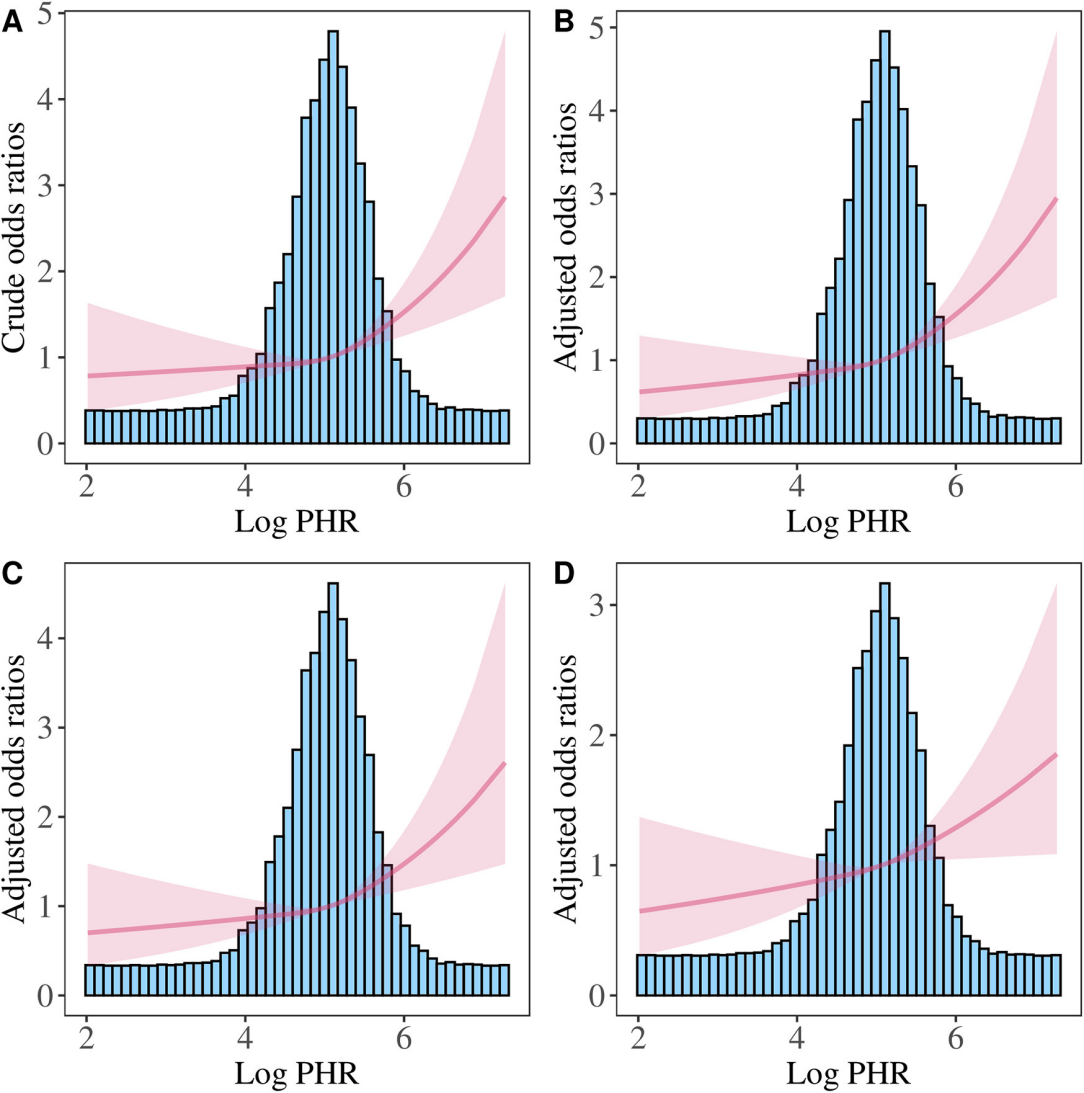
Restricted cubic spline analysis of the association between Log PHR and CVD risk. **(A)** Crude model: without adjusting for covariates. **(B)** Model 1: adjusted for age and sex. **(C)** Model 2: further adjusted for marital status, residence place, education level, smoking status, alcohol consumption TC, TG, and BMI. **(D)** Model 3: additionally adjusted for hypertension, diabetes, history of dyslipidemia medication use, chronic lung disease, chronic kidney disease.

**Figure 5:**
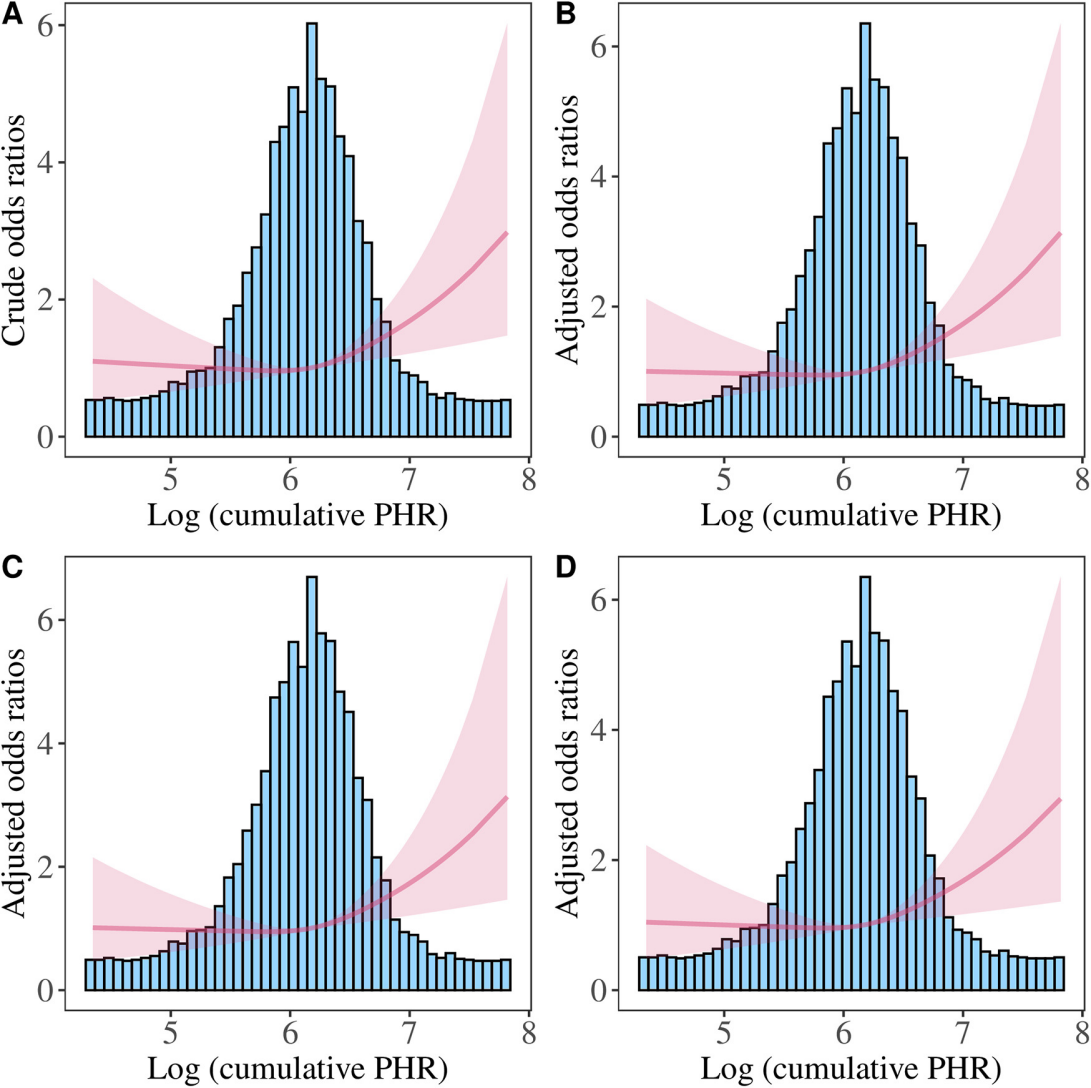
Restricted cubic spline analysis of the association between Log (cumulative PHR) and CVD risk. **(A)** Crude model: without adjusting for covariates. **(B)** Model 1: adjusted for age and sex. **(C)** Model 2: further adjusted for marital status, residence place, education level, smoking status, alcohol consumption, TC, TG, and BMI. **(D)** Model 3: additionally adjusted for hypertension, diabetes, history of dyslipidemia medication use, chronic lung disease, chronic kidney disease.

We further performed the RCS analysis, which revealed a significant overall association between Log PHR and CVD risk (*P* for overall <0.05) (Figure 4). However, there was no evidence of nonlinearity in this association (*P* for nonlinearity >0.05). The spline curve demonstrated a positive and graded linear association between Log PHR and CVD risk. Similar results were found when using Log cumulative PHR as the exposure (Figure 5).

### Subgroup analysis

**Figure 6:**
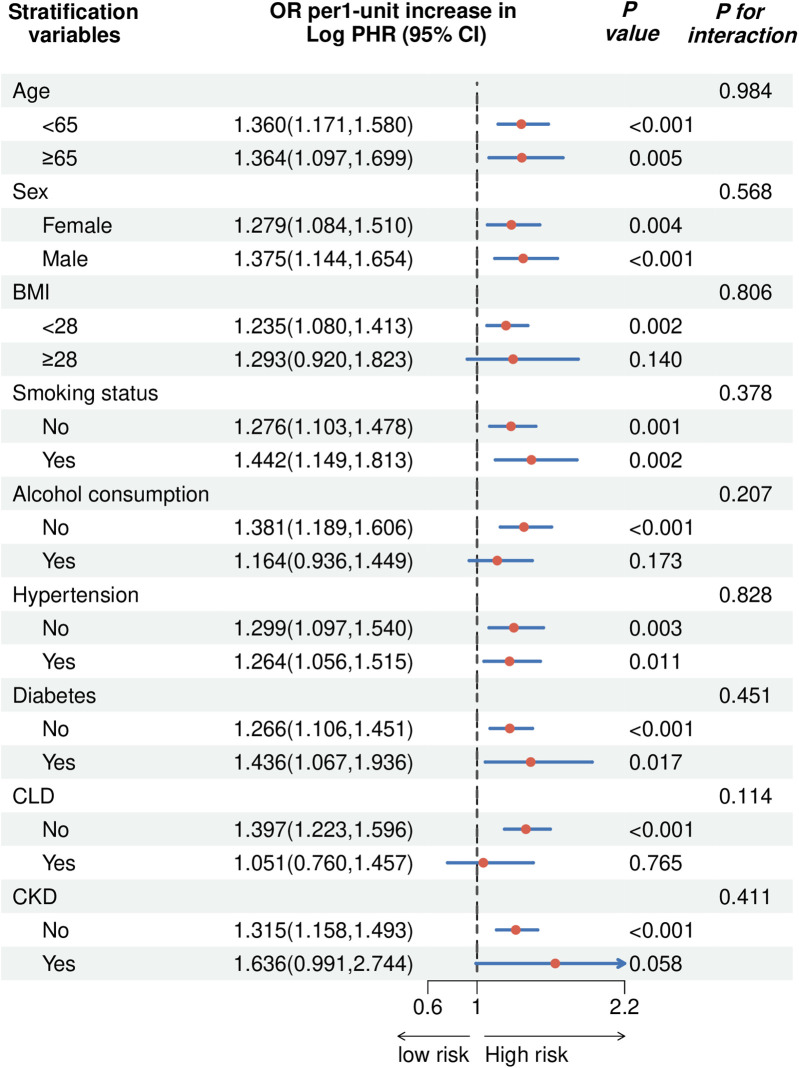
The forest plot showed the correlation between Log PHR and the risk of CVD (OR 95%).

Figure 6 presents the stratified analysis of the association between Log PHR and CVD across different subgroups, with OR and 95% CI displayed for each category. The association between Log PHR and CVD was significant in both age and sex groups. Among participants with BMI < 28, the association remained significant (OR: 1.235, 95% CI: 1.080–1.413, *P* = 0.002), whereas in those with BMI ≥ 28, the association was not statistically significant (OR: 1.293, 95% CI: 0.920–1.823, *P* = 0.140; *P* for interaction = 0.806). Both smokers (OR: 1.442, 95% CI: 1.149–1.813, *P* = 0.002) and non-smokers (OR: 1.276, 95% CI: 1.103–1.478, *P* = 0.001) exhibited a significant association (*P* for interaction = 0.378). For alcohol consumption, the association was significant among non-drinkers (OR: 1.381, 95% CI: 1.189–1.606, *P* < 0.001) but not among drinkers (OR: 1.164, 95% CI: 0.936–1.449, *P* = 0.173; *P* for interaction = 0.207). The association remained significant in both hypertensive (OR: 1.264, 95% CI: 1.056–1.515, *P* = 0.011) and non-hypertensive individuals (OR: 1.299, 95% CI: 1.097–1.540, *P* = 0.003; *P* for interaction = 0.828). Participants without diabetes had a significant association (OR: 1.266, 95% CI: 1.106–1.451, *P* < 0.001), while those with diabetes exhibited a stronger association (OR: 1.436, 95% CI: 1.067–1.936, *P* = 0.017; *P* for interaction = 0.451). In chronic lung disease (CLD) stratification, the association was significant in participants without CLD (OR: 1.397, 95% CI: 1.223–1.596, *P* < 0.001), but not in those with CLD (OR: 1.051, 95% CI: 0.760–1.457, *P* = 0.765; *P* for interaction = 0.114). For chronic kidney disease (CKD), the association was significant in individuals without CKD (OR: 1.315, 95% CI: 1.158–1.493, *P* < 0.001), but marginal in those with CKD (OR: 1.636, 95% CI: 0.991–2.744, *P* = 0.058; *P* for interaction = 0.411). Results of the subgroup analyses examining the relationship between cumulative PHR and CVD are shown in Supplementary Figure 1.

### Predictive ability of the PHR and cumulative PHR

The inclusion of Log PHR in the baseline model resulted in a slight improvement in the C-index (0.638, 95% CI: 0.622–0.654) compared to the baseline model alone (0.636, 95% CI: 0.620–0.652). Similarly, adding Log cumulative PHR improved the C-index (0.638, 95% CI: 0.613–0.663) compared to the baseline model (0.634, 95% CI: 0.609–0.660), though these differences did not reach statistical significance (Supplementary Tables S3, S4). However, the addition of Log PHR significantly improved risk classification, as indicated by an NRI of 0.1093 (*P* < 0.001) and an IDI of 0.0022 (*P* < 0.001). Likewise, incorporating Log cumulative PHR led to a significant NRI of 0.1163 (*P* = 0.011), though its IDI (0.0012) was only borderline significant (*P* = 0.053). While these findings suggest that Log PHR and Log cumulative PHR may enhance CVD risk stratification, their impact on overall model discrimination remains modest.

## Discussion

This large-scale prospective cohort study examined the association between the PHR and CVD risk in middle-aged and older adults in China. The findings indicated that an elevated Log PHR and Log cumulative PHR were significantly associated with an increased risk of CVD, even after adjusting for demographic, lifestyle, and clinical covariates. This association remained robust across most subgroups, and a significant dose-response relationship was observed, suggesting that PHR may serve as a novel biomarker integrating inflammatory and lipid-related pathways in the pathogenesis of CVD.

These results aligned with prior research highlighting the role of platelet-related and lipid-based markers in predicting CVD risk (25). Platelets contributed to thrombosis and inflammation, whereas HDL-C exerts cardioprotective, anti-inflammatory, and antioxidative effects (26, 27). The balance between these two components, reflected in PHR, provided additional insights into an individual's cardiovascular risk profile. While previous studies have examined platelet count (28, 29) and HDL-C (30, 31) separately in relation to CVD outcomes, few have investigated their combined impact through PHR and cumulative PHR. The current study extended existing knowledge by demonstrating the independent predictive value of PHR and cumulative PHR in a nationally representative cohort.

Several pathophysiological mechanisms may explain the observed association between higher PHR and increased CVD risk. Elevated platelet counts have been linked to platelet activation, endothelial dysfunction, and increased thrombus formation, all of which contributed to CVD development (32–34). HDL-C, known for its anti-inflammatory, antioxidative, and endothelial-protective properties, played a crucial role in cardiovascular health. Lower HDL-C levels may impair these protective functions, further promoting atherosclerosis (35–37). The cumulative PHR metric accounted for long-term exposure to an adverse platelet-lipid balance, reinforcing the importance of sustained inflammation and lipid dysfunction in CVD progression. These findings suggested that PHR may reflect both acute and chronic prothrombotic states, thereby improving risk stratification for cardiovascular events.

Despite the overall robust associations, certain findings from our subgroup analyses merit careful interpretation. The nonsignificant associations between PHR and CVD observed in some participant subgroups (e.g., those with obesity, chronic lung disease, or chronic kidney disease) might reflect a common underlying mechanism. These conditions share pathophysiological features characterized by low-grade chronic inflammation, which may alter both platelet reactivity and HDL functionality (38, 39). The systemic inflammatory state can enhance platelet activation while simultaneously modifying HDL composition and function (40, 41), potentially disrupting the conventional relationship between platelets and HDL-C that forms the basis of PHR's predictive utility. Previous studies have demonstrated altered platelet indices and lipid profiles in these conditions (42, 43), suggesting that the predictive value of PHR might need specific validation in populations with conditions that significantly affect platelet-HDL interactions.

In addition, although findings in the study may have potential clinical implications, the modest improvement in predictive performance metrics (NRI, IDI and C-index) warrants careful interpretation. While this increment may appear small in absolute terms, it is comparable to improvements typically observed in cardiovascular disease prediction when adding some established biomarkers to risk models (44–46). Previous research has demonstrated that even modest enhancements in discrimination indices can translate to meaningful clinical benefits, particularly for high-risk patient stratification. Importantly, PHR utilizes components (platelet count and HDL-c) that are routinely measured in many clinical settings. Unlike novel biomarkers requiring specialized testing, PHR can be derived from existing laboratory data without imposing significant additional costs. Clinically, identifying individuals at high risk for CVD remains a priority. The ease of calculating PHR from readily available laboratory tests, combined with its potential mechanistic significance in reflecting both inflammatory and lipid-related pathways, justifies further exploration of its clinical utility. Further studies are needed to explore how PHR can be effectively incorporated into clinical decision-making and whether it can guide personalized therapeutic interventions.

## Strengths and limitations

This study has several strengths. The use of a large, nationally representative cohort (CHARLS) enhanced the generalizability of the findings to middle-aged and older Chinese adults. Longitudinal study design allowed for the assessment of cumulative exposure to PHR, providing a more comprehensive understanding of its impact on CVD risk. The study employed robust statistical adjustments, controlling multiple confounders and validating the findings across different analytical models. Despite these strengths, the study has limitations. First, cardiovascular diseases in this study were ascertained through self-reported physician diagnoses, which could potentially introduce recall bias or diagnostic misclassification. To strengthen the validity of these findings, future research should incorporate clinically verified cardiovascular outcomes and medical record documentation. Second, although our method for calculating cumulative exposure has been used in previous CHARLS-based studies, the approach may oversimplify the temporal dynamics of exposure over time. Future studies with multiple measurements over time are needed to validate these findings. Third, although major CVD risk factors were adjusted, the possibility of residual confounding remains due to unmeasured factors such as genetic predisposition and dietary habits. Furthermore, the findings were derived from CHARLS participants aged 45 years and older, which may limit generalizability to younger populations with different risk factor profiles. Additionally, as the study population is predominantly Chinese, the results may not fully capture the genetic, regional, and environmental variations found in non-Asian or multi-ethnic populations. External validation in diverse cohorts is warranted.

## Conclusion

In conclusion, this study demonstrated that elevated Log PHR and Log cumulative PHR were significantly associated with an increased risk of CVD in middle-aged and older Chinese adults. This relationship was robust across multiple models and subgroups, indicating that PHR could be a potential biomarker for cardiovascular risk stratification. Given that PHR can be easily calculated from routine laboratory measurements, future research should focus on its clinical implementation and integration into existing risk assessment frameworks.

## Data Availability

The datasets presented in this study can be found in online repositories. The names of the repository/repositories and accession number(s) can be found below: http://charls.pku.edu.cn/.
