# Supplementary material for: Association of platelet-to-high-density lipoprotein cholesterol ratio and its cumulative exposure with cardiovascular disease risk: a prospective cohort study in Chinese population
Source: Front Cardiovasc Med. 2025 May 9;12:1580359. doi: 10.3389/fcvm.2025.1580359 (PMC12098545; doi:10.3389/fcvm.2025.1580359)

Supplementary Table S1. The results of the collinearity screening (Log PHR as independent variable).

Variables	VIF
Age	1.3
Sex	1.98
Marital status	1.11
Education level	1.47
Residence place	1.06
Smoking status	1.57
Alcohol consumption	1.32
TC	1.13
TG	1.2
BMI	1.09
Hypertension	1.1
Diabetes mellitus	1.08
Lipid-lowering drugs	1.04
Chronic lung disease	1.02
Chronic kidney disease	1.01

Supplementary Table S2. The results of the collinearity screening (Log cumulative PHR as independent variable).

Variables	VIF
Age	1.27
Sex	2.04
Marital status	1.1
Education level	1.44
Residence place	1.05
Smoking status	1.59
Alcohol consumption	1.34
TC	1.15
TG	1.21
BMI	1.09
Hypertension	1.1
Diabetes mellitus	1.08
Lipid-lowering drugs	1.04
Chronic lung disease	1.02
Chronic kidney disease	1.01

Supplementary Table S3. The C-index of the two model [(baseline model+Log PHR) vs baseline model], along with NRI and IDI analysis of the new model versus the baseline model in predicting CVD.

Reference Model	C-Index (95%CI)	C-Index (95%CI) of baseline model+Log PHR	*P* value for C-Index	NRI	*P* value for NRI	IDI	*P* value for IDI
baseline model	0.636 (0.620-0.652)	0.638 (0.622-0.654)	0.389	0.1093	<0.001	0.0022	<0.001

Supplementary Table S4. The C-index of the two model [baseline model+ Log (cumulative PHR) vs baseline model], along with NRI and IDI analysis of the new model versus the baseline model in predicting CVD.

Reference Model	C-Index (95%CI)	C-Index (95%CI) of baseline model+ Log (cumulative PHR)	*P* value for C-Index	NRI	*P* value for NRI	IDI	*P* value for IDI
baseline model	0.634 (0.609-0.0.660)	0.638 (0.613-0.663)	0.231	0.1163	0.011	0.0012	0.053

Supplementary figure 1. The Forest plot showed the correlation between Log (cumulative PHR) and the risk of CVD (OR 95%).

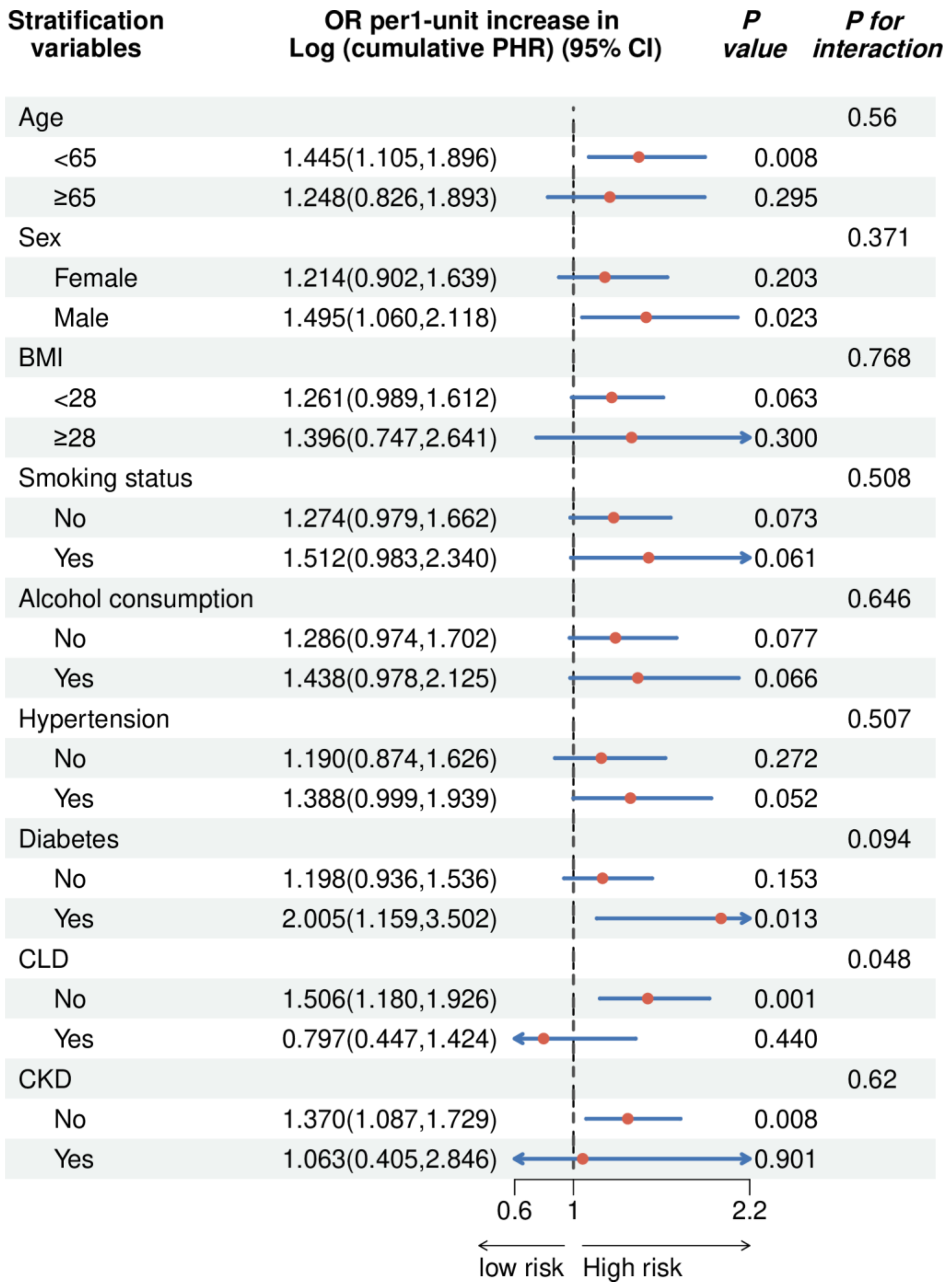

Supplement: Supplementary file 1 [file Datasheet1.pdf]
